# Oil Palm Empty Fruit Bunches as Raw Material of Dissolving Pulp for Viscose Rayon Fiber in Making Textile Products

**DOI:** 10.3390/polym14153208

**Published:** 2022-08-06

**Authors:** Siti Nikmatin, Irmansyah Irmansyah, Bambang Hermawan, Teddy Kardiansyah, Frederikus Tunjung Seta, Irma Nur Afiah, Rofiqul Umam

**Affiliations:** 1 Department of Physics, Faculty of Mathematics and Natural Sciences, IPB University, Bogor 16680, Indonesia; 2 Surfactant and Bioenergy Research Center, IPB University, Bogor 16143, Indonesia; 3 Center for Cellulose, Bandung Regency, Bandung 40258, Indonesia; 4 Faculty of Industrial Technology, Universitas Muslim Indonesia, Makassar 90231, Indonesia; 5 Graduate School of Science and Technology, Kwansei Gakuin University, Nishinomiya 669-1337, Hyogo, Japan

**Keywords:** oil palm empty fruit bunches, whole-empty fruit bunches, stalk-empty fruit bunches, dissolving pulp, viscose rayon fiber

## Abstract

The creative fashion industry produces several textile products that play an important role in the national economy. In various countries, this industry has continued to grow along with the strong flow of information technology and e-commerce. The development of textile products for fashion is very dynamic and competitive. Competition is not only about price, but also the quality of organic/synthetic materials, the comfort provided, and designs that change every 4–6 months. Recently, creative fashion not only relies on natural and synthetic polymer-made fibers but also biomass-based waste materials. Therefore, this study aims to manufacture textile products from biomass-based waste materials that can be applied to the creative fashion industry. Two types of raw materials from oil palm empty fruit bunches (EFB), namely, whole-empty fruit bunches (WEFB) and stalk-empty fruit bunches (SEFB), are used as an excellent innovation of rayon viscose fiber (RVF), a noncotton organic yarn capable of providing a solution to the 99% import of global cotton needs. This is expected to increase competitiveness, as well as the added value of palm oil products and their derivatives. The process of manufacturing DP chemically includes prehydrolysis, cooking, bleaching to dissolve the lignin and noncellulosic materials as well as isolation to purify POEFB fiber. Furthermore, DP testing is carried out to determine product quality and compare it with the national product standards. The results show that the alpha-cellulose content reaches >94% with variations in the active alkali of 18%, 20% and 24%. This implies that the WEFB and SEFB are used to fulfill the first requirements of the national standard (SNI 938:2017). The WEFB with an active alkali variation of 24% meets the SNI standard for rayon pulp with a value of S_10_ = 3.07 and S_18_ = 7.14%, while all variations of SEFB show opposite results. The use of active alkali at 24% had a brighter color than between 18% and 20%. Additionally, the fiber density of WEFB appears to be higher than that of SEFB. These results correlate positively with DP prepared using 24% alkali as the optimum treatment for all products, as well as the morphological observations performed with scanning electron microscopy (SEM), which shows that WEFB fixated fiber had a larger diameter than SEFB.

## 1. Introduction

One of the problems that arises with the growth of the creative fashion industry in Indonesia is the competitiveness of the world market for raw materials and processing technology [1]. The existing natural resources must be able to provide cotton or non-cotton materials for a diversity of fashion products [1,2,3]. According to several reports, the availability of local, semi-synthetic and cotton yarns is very limited. The national demand for cotton as a raw material for cotton textiles depends extensively on foreign supplies, with an estimated import value of 99.2% [2]. Therefore, efforts are needed to increase the amount and variety of local organic raw materials, namely, biomass from oil palm empty fruit bunches (EFB) in fashion products as an advanced and strategic innovation [4].

The development of biomaterials in the form of biomass from EFB in Indonesia is expanding along with the strong flow of the internet, entertainment, mass media and the textile business world [1,2,3]. Fashion is a lifestyle, and this creative industry ranks second, after culinary [2]. Indonesia’s dense population is an opportunity and an advantage in the absorption of the domestic fashion product market, while creativity, design, aesthetics and eastern culture create value to meet export demand [1,2].

**Figure 1:**
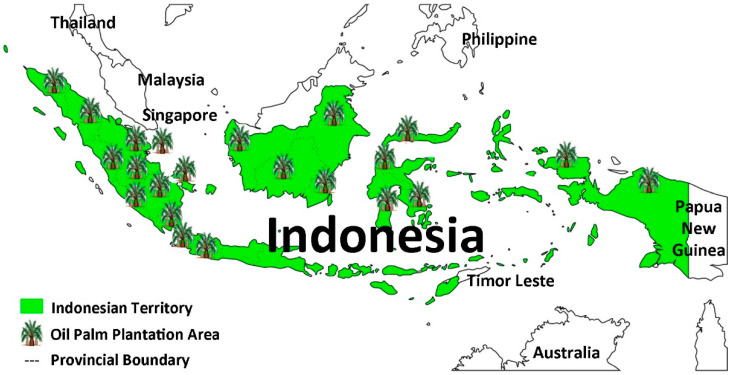
Palm oil plantations in Indonesia.

The area of oil palm plantations in Indonesia is approximately 15.08 million hectares (ha) as of 2021 and is spread across almost the entire islands, as illustrated in Figure 1. The problem of solid waste from crude palm oil (CPO) production, such as palm oil shells (POS), mesocarp fiber (MF) and empty fruit bunches (EFB). Especially EFB has the highest percentage, which is 65% of the total other solid waste [4], so it needs to be utilized in other new products and resolved through product diversification Processing of EFB into dissolving pulp (DP) can encourage the creation of new products and markets in textile products and sustainable national creative industries [5,6]. Additionally, the conversion into natural polymers in the form of synthetic cotton is a solution to overcome the low production and availability of natural fibers such as cotton, silk and wool [7].

The global market demand for dissolving pulp as the main ingredient for making rayon fiber is increasing every year. In Indonesia, it is estimated to be between 400,000 and 500,000 tons per year and will continue to increase in line with the growth of the rayon fiber industry [3,4,5]. The consumption of RVF controls 80% of the market share of cellulose fibers, and the growth of viscose production for the textile industry is increasing to replace polyester and cotton. The main factors driving the high demand are economic growth, the population and demand in the medical and health sectors. Currently, only about 2.5% of the world’s agricultural land is planted with cotton, and this will not meet the demand in the future [6,7]. Low productivity and availability as well as high demand make cotton very expensive and extensively imported. Meanwhile, synthetic fibers in textile applications such as polyester, nylon and spandex have an environmental impact due to the use of non-biodegradable, non-renewable resources and increasing carbon emissions [8]. This promotes the search for alternative raw material solutions as a substitute for petroleum-based polymer fibers and to meet the needs of non-cotton textile fibers with sustainable palm biomass through natural polymer-based cellulose. This study was conducted to convert biomass from EFB into DP as a raw material for rayon viscose fiber (RVF) in textile and fashion applications [9].

In terminological nature, fibers (grain) and texture are physical properties of wood structures that have a relationship with the drying process. The terms “fine grain” and “coarse grain” refer to the relationship with the growth circle pattern or growth circle width. However, when it comes to intercellular relationships, the term “fiber refers” to the longitudinal direction of cells or wood fibers. Therefore, straight fibers are defined as fibers that are perpendicular to the longitudinal plane, while inclined and twisted/spiral fibers are defined as the direction of the fibers forming the longitudinal plane [5,6].

The texture of the fiber in plants refers to the size of the cells. Fine texture refers to plants that have tree trunks with small diameters, while coarse-textured fibers are obtained from wood with large diameters. For cells that are almost the same size, the texture is called uniform [9]. To obtain fiber with the desired criteria, a biomaterial engineering process is needed by mixing other materials.

This biomaterial engineering is expected to produce local non-cotton raw materials from a variety of natural resources in Indonesia that are environmentally friendly in fashion applications. The results obtained are also expected to produce innovative substitute products for sustainable palm-based fashion to reduce imported raw materials in the form of synthetic fibers such as polyester and rayon made from cotton [10,11,12]. This will contribute immensely through science and technology to provide solutions to two national problems, namely, the abundance of EFB waste and imports of raw materials for the creative fashion industry. Consequently, government programs for import substitution and export orientation to strengthen the competitiveness of local creative industries from upstream to downstream can be achieved.

## 2. Materials and Methods

In this study, we compared the fiber produced from the different types of palm oil used, namely, whole-empty fruit bunches (WEFB) and stalk-empty fruit bunches (SEFB). When compared with previous studies, no one has compared the results of WEFB and SEFB fibers by adding active alkali variations as an ingredient to obtain better results. The manufacture of biomass-based dissolving pulp (DP) from oil palm empty fruit bunches (EFB) was carried out in 5 stages, namely, (1) synthesis of EFB long fibers by in-situ retting mechanical method; (2) pre-hydrolysis [13]; (3) fiber ripening; (4) dissolving pulp bleaching; (5) pulp dissolving test.

**Figure 2:**
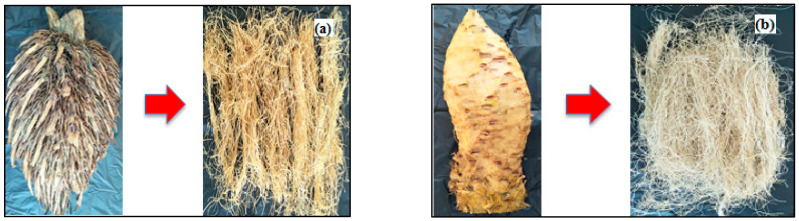
(**a**). WEFB is converted into fibers with a length of 10–20 cm before the cutting process with a cutting machine; (**b**). SEFB is converted into fibers with a length of 10–30 cm before the cutting process with a cutting machine.

The main material used was 6 tons of EFB obtained from PTPN VIII Cikasungka Bogor, Indonesia. It was divided into 2 types, namely, whole-empty fruit bunches (WEFB) as sample 1 and stalk-empty fruit bunches (SEFB) as sample 2 (Figure 2). Both were used as DP materials. A special processing technique was used to convert WEFB and SEFB into long fibers, which were then cut using a cutting machine and reprocessed to obtain DP. The processing of EFB from the beginning into long fiber was carried out with the Muda Manunggal Alam (MMA) farmer group in the Wirajaya oil palm plantation village area, Bogor district, West Java, Indonesia.

### 2.1. Preparation Stage

The first sample was 2 tons of whole-empty fruit bunches (WEFB), which were crushed using a crusher/milling machine at a continuous capacity of 100 kg/h to break down into long fibers measuring 10–20 cm. Subsequently, the fibers were washed with dynamic fluid (H_2_O) in 3 cycles in an open system soaking bath to remove impurities, and then air dried to obtain a uniform moisture content.

**Figure 3:**
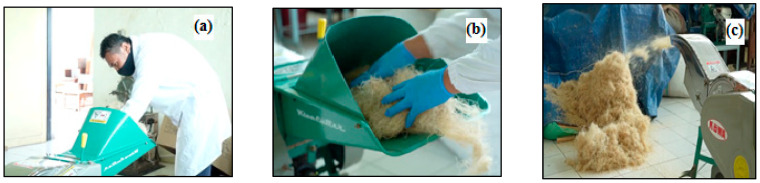
(**a**). WEFB fiber and SEFB fiber are cut using a cutting machine; (**b**). The cutting process is performed manually slowly to obtain good cutting results; (**c**). The long fibers that have been cut have a size of about 2–3 cm.

The second sample, namely, 4 tons of stalk-empty fruit bunches (SEFB) was processed with a mechanical method and soaked until long fibers up to 30 cm in size were formed. Furthermore, both WEFB and SEFB fibers were air dried to obtain uniform moisture content using the standard oven method [14]. The two samples were cut using a cutting machine to a size of 2–3 cm as shown in Figure 3. For synthesis and milling with a size of 60 mesh, chemical raw materials (type/brand) were used, namely, (1). Alpha cellulose ASTM D 1103; (2) Holocellulose ASTM D1104–56; (3) Extractive SNI 8401; (4) Lignin SNI 8429; (5) Pentosan SNI 01-1561; (6) Ash content SNI ISO 1762; (7) Solubility in cold water SNI 01-1305; (8) Hot water solubility SNI 01-1305; (9) 1% NaOH solubility SNI 14-1838.

### 2.2. Pre-Hydrolysis Stage

**Figure 4:**
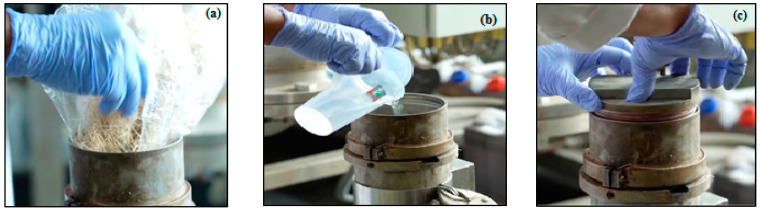
(**a**). EFB fiber (first sample and second sample) with a length of 2–3 cm and a mass of 300 g was inserted into the rotary digester tube (**b**). Addition of 1 N sulfuric acid, 0.4% sulfate pre-hydrolysis with a ratio of 1:5 (*w*/*v*); (**c**). EFB fiber was cooked at 130 °C for 60 min (time to reach maximum temperature was 60 min and held for 90 min).

Pre-hydrolysis of kraft was carried out on the first and second samples to determine the total yield and filtered yield. EFB fibers with a length of 2–3 cm, a mass of 300 g oven-dried with a moisture content of <10.32% were then placed into a rotary digester tube, with 4 replications, while 1 N sulfuric acid (equivalent number per mole), sulphate 0.4% with a ratio of 1:5 (*w*/*v*) was added. The fibers were cooked at 130 °C for 60 min, the time to reach maximum temperature was 60 min and held for 90 min [13]. Subsequently, the sample was removed from the tube, separated from the prehydrolysis liquor, and then washed with water until neutral. The pre-hydrolysis process and the synthesis conditions are shown in Figure 4 and Table 1.

### 2.3. Cooking Process

**Figure 5:**
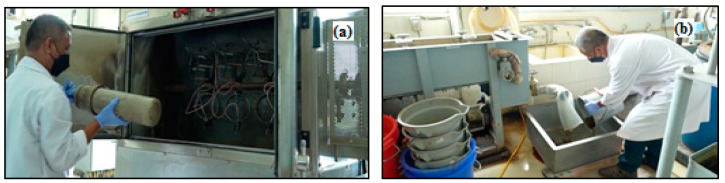
(**a**). The pulp from the first stage was put into the CRS AB rotary digester reactor with a capacity of 6 L and cooked at a temperature of 165 °C for 60 min. To reach the maximum temperature the pulp is held for 90 min, hence, the total time in the cooking process is 150 min; (**b**). The cooked pulp is then separated from the black liquor, washed and then screened to separate it from the residue.

The cooking process is the next stage after pre-hydrolysis. The pulp produced from the first stage is placed into the CRS AB rotary digester reactor with a capacity of 6 L. Subsequently, it was cooked at a temperature of 165 °C for 60 min to reach the maximum temperature and held for 90 min, hence, the total time was 150 min with a factor of H or delignification rate of 988.6. The amount of active alkali was varied at 18%, 20% and 24% for samples a, b, and c, respectively, on WEFB as a base reference with a sulfidity of 30% at a ratio of 1:5 (*w*/*v*), while the target Kappa number ranges from 14 to 18. The pulp was separated from the black liquor, washed and then screened to be separated from the residue. The yield was calculated based on the oven-dry weight of the pulp that passed the sieve divided by that of the raw materials (Table 2 and Figure 5).

### 2.4. Bleaching

**Figure 6:**
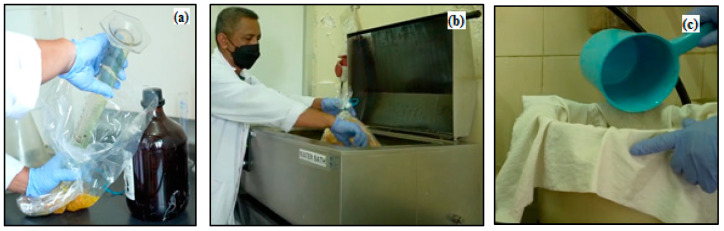
(**a**). The initial chlorine dioxide addition process is 0.22 KN (kappa number); (**b**). Heating with a time of 30 min and a temperature of 95 °C; (**c**). EFB fiber was cooked at 130 °C for 60 min (time to reach maximum temperature was 60 min and held for 90 min).

This is a step in producing the characterization of dissolving pulp according to the SNI 0938: 2017 standard through a bleaching sequence. The complete process of each step is presented in Figure 6. The dosage of ClO_2_ at D_0_ was calculated based on the Kappa number when the pulp is not yet white. Bleaching of WEFB samples with active alkali 18, 20 and 24% was carried out with the bleaching steps D_0_ – E_p_ – D_1_ – D_2_ and acids as described in Table 3. D_0_ is a treatment implemented by adding initial chlorine dioxide of 0.22 KN (kappa number) with a time of 30 min and a temperature of 95 °C. Meanwhile, Ep is an extraction treatment with peroxide (H_2_O_2_) 1% by adding 1% NaOH for 60 min at a process temperature of 70 °C, while in sample 2, the bleaching stage was carried out at 24% active alkali.

D_1_ is the step of adding 1% chlorine dioxide (ClO_2_) for 180 min at a temperature of 75 °C, while in D_2,_ 0.5% chlorine dioxide is further added for 180 min at a temperature of 75 °C. Stage O involves delignification of oxygen 87 psig by adding 1.5% NaOH for 30 min at a temperature of 95 °C in the sample concentration of active alkali 18% and 20%. Meanwhile, samples with a concentration of 24% were treated with 0.5% sulfuric acid (H_2_SO_4_) for 5 min at room temperature.

### 2.5. Dissolving Pulp (DP)

**Figure 7:**
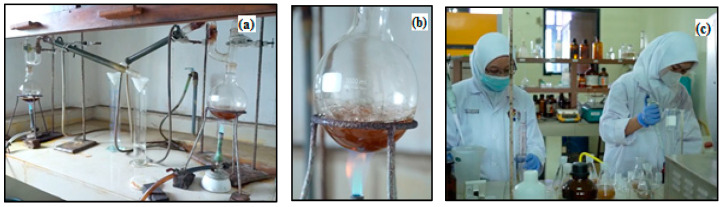
(**a**). SNI-Pulp Rayon; (**b**). Round bottom flask; (**c**). EFB pulp dissolving test process.

The dissolving pulp was tested according to SNI-Pulp Rayon as demonstrated in Figure 7. The parameters tested are as follows: (1) SNI 7070 water content; (2) ash content of SNI 0442; (3) silica/acid insoluble ash SNI ISO 776; (4) SNI 8400 alpha-cellulose; (5) S18 SNI ISO 692; (6) S10 SNI ISO 692; (7) SNI 8402 viscosity; (8) viscosity limit (intrinsic viscosity) SNI ISO 5351; (9) Ca content of TAPPI T 266; (10) Fe content TAPPI T 266; (11) degree of brightness SNI ISO 2470-1; (12) extractive SNI 8401.

## 3. Results

### 3.1. Analysis Results of Empty Fruit Bunches (EFB)

The yield and quality of twisted yarn obtained from WEFB and SEFB were affected by the type and preparation of raw materials, cooking processes and bleaching (Table 4). Product pictures starting from EFB processing into twisted yarn are shown in Figure 2. The raw material was prepared using a two-step method, namely, mechanical fibrillation, as well as retting and dynamic fluid washing in an open system (Figure 4, Figure 5 and Figure 6). It aims to remove impurities, thereby increasing alpha-cellulose, as well as reducing the ash content and the use of chemicals in bleaching to improve the quality of twisted yarn and dissolving pulp (DP) produced [13]. Table 4 shows the chemical testing results of WEFB and SEFB treatments, which can be used describe the effect of raw material preparation on the quality of the long fiber produced.

Based on the compositional analysis results, SEFB parameters of moisture and ash content, pentosan, extractives, lignin, holocellulose and alpha-cellulose are better compared to WEFB fiber and meet the criteria for DP based on references/citations of artificial fiber materials [15,16]. Experimental results showed that the preparation and pre-hydrolysis steps can increase the efficiency of mechanical defibrillation in the extraction process of non-fibrillated cellulose by removing impurities and destroying the cell wall structure [17,18,19,20]. This is an appropriate first step for further chemical cooking processes because cellulose is composed of linear chains of glucose polymers and forms supramolecular structures that are strongly hydrogen-bonded; hence, they are not easily soluble in water and ordinary organic solvents [18].

Compared to WEFB, SEFB fiber has a larger average length but a smaller diameter. This is linear with the results of the raw material readiness test (Table 4), which demonstrated a decrease in non-cellulose components but an increase in alpha-cellulose [21]. Meanwhile, the dimensions and fiber ratios such as fiber length and diameter are used to determine the quality of the raw materials for twisted yarn and pulp produced [13]. The average WEFB fiber length was 144.70 ± 25.92 mm with a diameter of 0.29 ± 0.07 mm, while the average SEFB length was 242.30 ± 70.95 mm with a diameter of 0.28 ± 0.06 mm. The density of both materials was 0.29 ± 0.06 g/cm^3^ and 0.71 g/cm^3^, respectively (Table 5).

### 3.2. Dissolving Pulp (DP) and Rayon Viscose Fiber (RVF)

Generally, textile raw materials are made from natural and artificial fibers or a combination of both. Natural fibers are obtained from plants or animals, such as cotton, wool, linen and silk [6,7], while those made by humans are referred to as artificial or synthetic [22]. Fibers as textile raw materials are classified into three groups based on their sources, namely, natural polymers comprising rayon viscose, carbamate and lyocell; synthetics such as polyester, nylon, acrylic and spandex; as well as those from inorganic materials including metal fibers [23]. Natural fibers are more convenient to use, environmentally friendly, expensive and require sustainable cultivation, while the artificial types have a low price, are more durable, high production, are inorganic and cannot be decomposed naturally, but their production involves processes that threaten the environment and human health. One of the high-quality textile materials is rayon viscose fiber (RVF) [24]. However, RVF is included in the type of semisynthetic fiber. Semisynthetic fibers are derived from natural fibers but are manufactured through a chemical process. So basically, natural fibers are harvested, broken down, and then reconstructed to obtain the final product in the form of cellulose. Cellulose is an abundant structural component in plants, which is then extracted, made soluble and then spun into fiber. Some researchers carry out this process, because in a sustainable manner, semisynthetic fibers can produce very versatile fabrics [20,21,22,23,24].

Rayon viscose fiber (RVF) is a type of semisynthetic fiber made from the regeneration process of cellulose, which can be obtained from hardwood, softwood and non-wood. The content in these raw materials is processed into a special pulp, which has certain characteristics, namely, alpha-cellulose, high brightness [10,11,12,13], extractive content, hemicellulose, low ash and better resistance to alkaline solutions [25,26,27]. This is called dissolving pulp, and 70% of its application is to manufacture cellulose fibers through the viscose process [6,7,8]. The alpha-cellulose content in the dissolving pulp must be higher than 94% [10], while the hemicellulose is in the range of 2–4% and other requirements in the Indonesian National Standard (SNI) are as shown in Table 6. The DP has advantages over paper pulp, namely, high cellulose reactivity and uniform molecular weight distribution [11]. The production of EFB involves sulfuric acid with a low concentration and added cooking solutions, namely, NaOH and Na_2_S. The use of NaOH serves to degrade and dissolve lignin for easy separation from the fiber. Meanwhile, Na_2_S functions to maintain carbohydrates especially cellulose from degradation for high yields and good physical strength [6,7,8,9,10].

Alpha cellulose content of DP >94% on WEFB indicates the fulfillment of the SNI 938:2017-DP requirements on variations of active alkali of 18% and 24% (Table 7). Dissolving pulp with a high content can produce high yields [28,29,30] and is very suitable as a raw material for making viscose rayon fiber [6,7,8,9]. The solubility in sodium hydroxide of 10% (S_10_) and 18% (S_18_) indicates the purity of DP. The lower solubility indicates higher purity. The 10% sodium hydroxide dissolved hemicellulose and short-chain cellulose, namely, polymers with a molecular weight of less than 25,000 g/mol. Meanwhile, the 18% sodium hydroxide only dissolved hemicelluloses with a molecular weight of less than 8000 g/mol [31]. Based on the results, the WEFB with an active alkali of 24% met the standard of SNI938:2017 DP with a value of S_10_ = 3.07 and S_18_ = 7.14% (Table 8).

The dissolving pulp (DP) analysis results are listed in Table 8. The low intrinsic viscosity of 331.80 mL/g needs to be increased to >370 through the curing step [32]. Meanwhile, the high ash content of 1.80% was due to the insufficient initial pre-treatment of the raw material with 3 cycles of washing; hence, decortication is required before the pre-hydrolysis process [13]. During this process, mineral substances are released from the material due to mechanical treatment. Extractive levels of 0.82%, Ca 608 mg/kg, and Fe 354 mg/kg (Table 8) can affect the further processing of viscose rayon. This implies that there is a need to add surfactants during the cooking process [33] and at the extraction stage of bleaching [34,35,36]. The extractive and ash content of the raw materials tend to cause problems such as difficulty in handling black leachate, build-up, equipment breakdown, pipe blockage, pitch problems and especially DP quality degradation.

Solubility in cold and hot water, as well as 1% NaOH, reflects the extractive content, hemicellulose and degraded cellulose (Table 4). EFB has a high starch content, culminating in a lower pulp yield, as shown in Table 8 [37]. The yield of cooking on three variations of active alkali showed 33–40% of the raw material on pre-hydrolysis [13]. Furthermore, the kappa number was used to measure the quality of the pulp. It is defined as the amount of lignin residue after cooking and indicates the pulp’s ability to bleach, controls cooking and determines the chemical requirements for the bleaching process [10,11,12]. The variation in the kappa number obtained was due to the lignin content in the sample. The greater the number, the higher the lignin content remaining in the pulp and the greater the amount of bleach that will be used in the bleaching process. Table 8 shows that the sample with the alkali variation of 24% had a value of 11.24%, with the highest brightness level of 85% and the final bleaching yield of 32%.

Dissolving pulp (DP) with bleaching sequence D_0_E_p_D_1_D_2_ in active alkaline samples of 18 and 20% had final yields of 37.29 and 36.49%, with a brightness of 82 and 81%, respectively. Meanwhile, the 24% active alkali sample with the sequence D_0_E_p_D_1_D_2_ and added with peroxide (H_2_O_2)_ had a yield of 32.31% with a brightness of 85%. This implies that bleaching with different processes showed different yields and degrees of brightness. The sequence D_0_E_p_D_1_D_2_ generated DP with a high yield but low brightness, as shown in Table 7 and Table 8. The hydrogen peroxide in the active alkaline sample of 24% increased the brightness of the pulp but reduced the yield due to its non-selective degradation, which attacks lignin and cellulose [37].

Table 7 shows that WEFB with the use of 24% active alkali produced optimal results based on the water content, alpha-cellulose, brightness and kappa number. The sample meets the Indonesian National Standard (SNI 938:2017) for pulp dissolving analysis; hence, a pulping test was only carried out using this concentration as shown in Table 8. The results of WEFB with the use of active alkali 24% can be applied as a reference for SEFB. However, the 18% sample in WEFB had alpha-cellulose, which meets the SNI 983:2017 standard. The subsequent treatment given to SEFB was the addition of an active alkali variation of 18%, 20% and 24% (Table 9).

**Figure 8:**
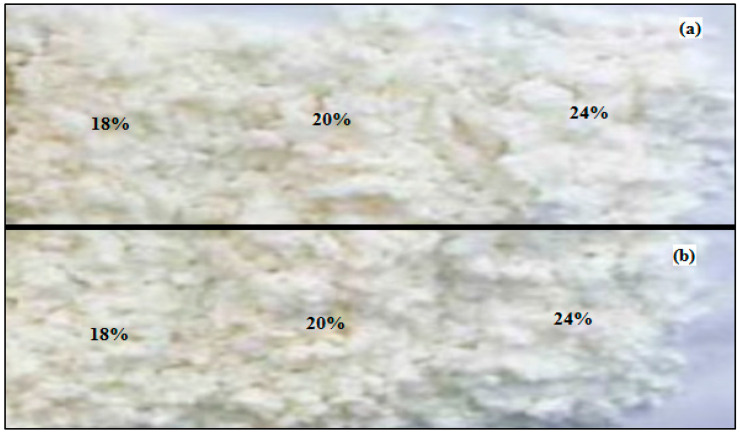
(**a**). Differences in brightness and density of WEFB fiber pulp with active alkali variations of 18%, 20% and 24%; (**b**). Differences in brightness and density of SEFB fiber pulp with active alkali variations of 18%, 20% and 24%.

**Figure 9:**
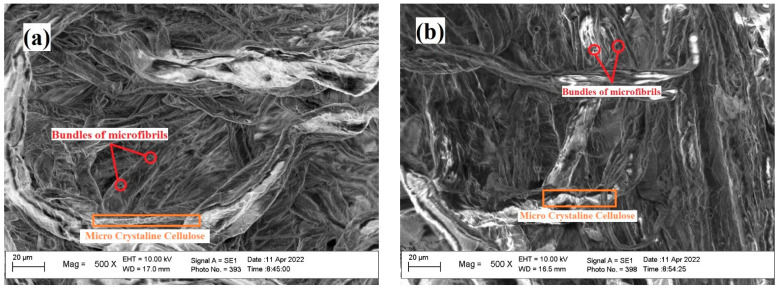
(**a**). Morphological observations using scanning electron microscopy (SEM) on WEFB viscose fibers; (**b**). Morphological observations using SEM on SEFB viscose fibers.

The brightness level of each DP generated from WEFB and SEFB was not significantly different as shown in Figure 8. However, the results show that the use of active alkali at 24% has a brighter color than at 18% and 20%. The density level of WEFB was also higher compared to that of SEFB. These results also have a positive correlation with morphological observations performed using scanning electron microscopy (SEM) with a magnification of 500× in Figure 9.

The morphological observation results showed that RVF from WEFB biomass had a larger diameter than SEFB with values of 17.00 mm and 16.5 mm, respectively (Figure 9). In addition, SEM micrographs show the surface morphology of WEFB fibers consisting of bundles of microfibrils with deposited cellulose components such as wax, lignin and hemicellulose [38,39,40]. The aggregation of micro crystals observed is presumably due to the water evaporation step during sample preparation. Aggregation of micro-crystalline cellulose (MCC) can be produced by strong hydrogen bonds between MCC and also by the drying method. The micrograph also shows rod-shaped MCC, which all had a diameter of less than 50 nm.

### 3.3. Chemical Characteristics of EFB Using Fourier Transform Infrared Spectroscopy (FTIR)

A previous study by Harsono et al. 2016 [13] reported that EFB contains glucan (cellulose), xylan (hemicellulose) and lignin, comprising acid insoluble and soluble with percentages of 35.7%, 20.1% and 27.6%, respectively. Although the glucan and xylan content is lower than bagasse, previous investigations confirmed that EFB can be used for the production of paper and cardboard. This indicates that EFB is also very suitable as a basic material for textile fibers [41].

**Figure 10:**
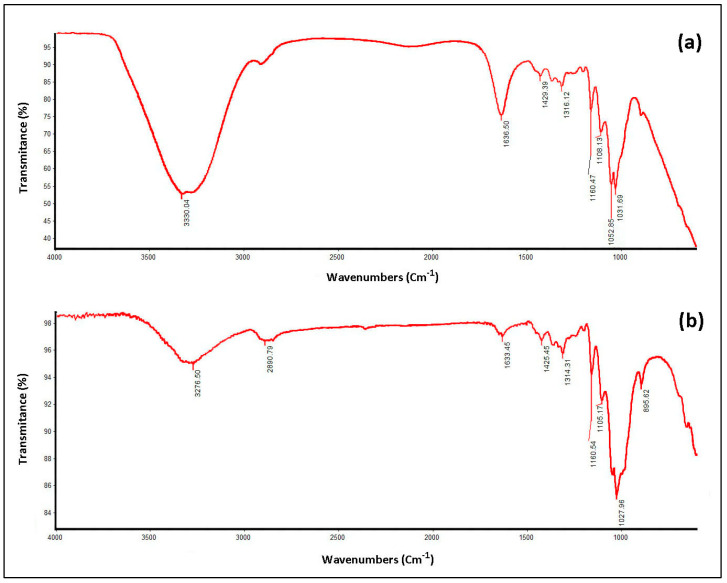
Fourier transform infrared spectra of the EFB (**a**) WEFB fibers and (**b**) SEFB fibers.

Chemical characteristics observed using the FTIR spectrum (Figure 10) show that the bandwidth of between 3276.50 cm^−1^ and 3330.04 cm^−1^ represents strong intermolecular bonding forces from stretching vibrations between abundant phenolic and aliphatic hydroxyl in lignin extracted from SEFB (Figure 10b) and WEFB (Figure 10a). Spectrum 2890.79 cm^−1^ in each RVF is lignin from the stretching vibration of the CH methyl group. In addition, the presence of stretching vibrations for the alkene group was found in the spectrum at 1633.45 cm^−1^ from SEFB (Figure 10b) and 1636.50 cm^−1^ from WEFB, while aromatic skeletal vibrations were indicated by 1425.45 cm^−1^–1429.39 cm^−1^. A phenylpropane signal was detected in the lignin structure on strain vibration between 1314.31 cm^−1^ and 1316.12 cm^−1^ for CO aromatic ester. Hardwood lignin contains a mixture of guaiacyl (G) and syringyl (S); hence, the spectrum in each sample (Figure 10) shows the stretching vibration of CO in syringyl (S) units around 1160.47 cm^−1^ in WEFB and 1160.54 cm^−1^ in SEFB. Moreover, two adjacent signals at 1105.17 cm^−1^ and 1108.13 cm^−1^ associated with the deformation of the ester bond and the presence of a guaiacyl (G) structure were also present [42,43].

### 3.4. Chemical Characteristics of EFB Using X-ray Diffraction (XRD)

**Figure 11:**
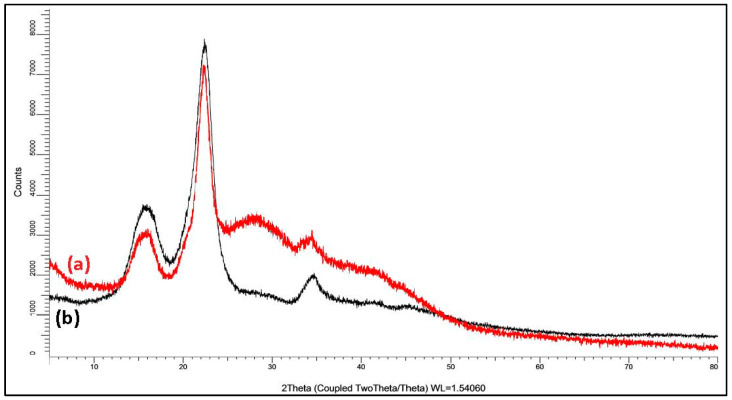
X-ray diffraction patterns of dissolving pulp (**a**) whole-empty fruit bunches (WEFB) and (**b**) stalk-empty fruit bunches (SEFB).

The crystallinity index (CrI) of all samples was calculated based on the XRD spectra of DP WEFB and SEFB. Using the field 200 on 2θ = 23° in all asymmetric samples, the CrI increased with rising temperature. Crude WEFB had the lowest value, namely, 41.83%, influenced by the presence of lignocellulose in the EFB structure. Besides, the residual fibers obtained after lignin extraction were characterized, as depicted in Figure 9, Figure 10 and Figure 11. SEFB undergoes reactions at high temperatures with a high degree of crystallinity, up to 78%. The crystallinity index of each sample is different due to the amorphous or crystalline structure, indicating that there is a fairly high amount of lignin removal [44,45]. All diffraction patterns showed peaks around 2θ = 16°, 22.5° and 35° with a characteristic cellulose I structure. The XRD peaks of dissolving pulp indicate the remaining MCC after the treatment.

## 4. Conclusions

Based on the results, the yield and quality of twisted yarn obtained from whole-empty fruit bunches (WEFB) and stalk-empty fruit bunches (SEFB) are influenced by the type and preparation of raw materials, cooking process and bleaching. The composition analysis results showed that the SEFB parameters of moisture and ash content, pentosan, extractive, lignin, holocellulose and alpha-cellulose were better than WEFB fiber and met the criteria for DP based on references/citations for artificial fiber materials. The preparation and pre-hydrolysis steps tend to increase the efficiency of mechanical defibrillation in the extraction process of non-fibrillated cellulose by removing impurities and destroying the cell wall structure. Compared to WEFB, SEFB fibers have a larger average length but smaller diameter. This is linear with the readiness test results, which showed a decrease in non-cellulose components and an increase in alpha-cellulose. Meanwhile, the dimensions and ratios such as length and diameter are used to determine the quality of the raw material for twisted yarn and pulp produced. The alpha-cellulose content of DP >94% in WEFB fulfills the requirements of SNI 938:2017-DP at a variation of 18% active alkali and 24%. According to previous studies, dissolving pulp (DP) with a high content can produce significant yields. Solubility in sodium hydroxide is 10% (S_10_) and 18% (S_18_) reflects the purity. The lower the solubility, the higher the purity. The 10% sodium hydroxide dissolved hemicellulose and short-chain cellulose, namely, polymers with a molecular weight of less than 25,000 g/mol, while 18% only dissolved hemicelluloses with a molecular weight of less than 8000 g/mol. Furthermore, WEFB on the variation of active alkali at 24% met the SNI938:2017 DP standard with a value of S_10_ = 3.07 and S_18_ = 7.14%. These results indicate optimum values and can be used as a reference for SEFB, but the use of 18% active alkali in WEFB had alpha-cellulose that meets the SNI 983:2017 standard. The subsequent treatment given to the SEFB was the addition of an active alkali variation of 18%, 20% and 24%. The brightness level of each DP produced from both samples showed no significant difference. However, the results indicate that the use of active alkali 24% had a brighter color than at 18% and 20%. The micro crystals’ aggregation was also observed in the micrograph due to the water evaporation step during sample preparation. The aggregation of micro crystalline cellulose (MCC) can be produced by strong hydrogen bonds between MCC and also by the drying method. The micrograph shows rod-shaped MCCs, which all had a diameter of less than 50 nm. The chemical characteristics observed using the FTIR spectrum show that the broad band between 3276.50 cm^−1^ and 3330.04 cm^−1^ represents strong intermolecular bonding forces from stretching vibrations between the abundant phenolic and aliphatic hydroxyl in lignin extracted from SEFB. Furthermore, the crystallinity index (CrI) of all samples was calculated based on the XRD spectra. According to the 200 fields at 2θ = 23° in all asymmetric samples, the CrI of each sample increased with rising temperature. The crude WEFB had the lowest value, which was 41.83%, due to the presence of lignocellulose in the EFB structure. The XRD peaks of the dissolving pulp indicated the remaining MCC after the treatment.

## Figures and Tables

**Table 1:** Raw materials and pre-hydrolysis process conditions for whole-empty fruit bunches (WEFB) and stalk-empty fruit bunches (SEFB).

**No**	**Component**	**Condition**	
1	Cooking sequence number	102	104
2	Code raw material	WEFB	SEFB
3	Cooking No.	102/EFB/21	104/EFB/21
4	Process	Pre-hydrolysis	Pre-hydrolysis
**No**	**Component**	**Raw material**	
1	Name of raw material	WEFB	SEFB
2	Moisture content (%)	10.32	10.32
**No**	**Component**	**Material chemical (H2SO4)**	
1	Cons, H2SO4 (N)	1.0	1.0
**No**	**Component**	**Weight of raw material and pre-hydrolysis conditions**	
1	Dry weight (grams)	300	300
2	Ratio (*w*/*v*)	5	5
3	H2SO4 (%)	0.40	0.40
4	Temperature (°C)	130	130
5	Destination time (minutes)	60.0	60.0
6	Time at (minutes)	60.0	60.0
7	Factor H	988.6	988.6
**No**	**Component**	**Raw materials and pre-hydrolysis chemicals**	
1	Wet weight (grams)	334.52	334.52
2	Water in chip	34.52	34.52
3	H2SO4 (mL)	24.59	24.59
4	Addition of water (mL)	1.441	1.441

Source: Data of this study.

**Table 2:** Raw materials and ripening conditions for pulp of whole-empty fruit bunches (WEFB) and stalk-empty fruit bunches (SEFB).

**No**	**Component**	**Condition**	
1	Cooking sequence number	102/WEFB/21	104/SEFB/21
2	Process	Kraft	Kraft
**No**	**Component**	**Raw material**	
1	Name of raw material	WEFB	SEFB
2	Moisture content	62.4	60.1
3	Weight after pre-hydrolysis/refening	798.10	752.00
**No**	**Component**	**Chemicals (NaOH and Na_2_S)**	
1	Cons. NaOH (grams/L)	336.19	336.19
2	Dens. NaOH (grams/mL)	1.3	1.3
3	Cons. Na_2_S (grams/L)	107.29	107.29
4	Dens. Na_2_S (gram/mL)	1.16	1.16
**No**	**Component**	**Weight of raw materials and cooking conditions**	
1	Dry weight (grams)	300.0	300.0
2	A.A, NaOH (%)	20.00	20.00
3	Sulfidity (%)	30.00	30.00
4	NaOH (%)	18.06	18.06
5	Na_2_S (%)	7.55	7.55
6	Ratio (*w*/*v*)	5.00	5.00
7	Temperature (°C)	165.00	165.00
8	time (minutes)	60.00	60.00
9	Time on (minutes)	90.00	90.00
10	Factor H	988	988
**No**	**Component**	**Material requirements Raw materials and cooking chemicals**	
1	Wet weight (grams)	798.10	752.00
2	of NaOH (mL)	161.20	193.44
3	Weight of NaOH solution (grams)	209.56	251.47
4	Volume of Na_2_S (mL)	211.06	253.28
5	Weight of Na_2_S solution (grams)	244.84	293.80
6	Volume of water in standard ingredients (grams)	498.10	452.00
7	Weight of solution (grams)	1500.00	1500.00
8	Addition of water (mL)	547.51	502.73

Source: Data of this study.

**Table 3:** Process bleaching is carried out in 6 stages on several samples.

Stage	Treatment	Active Alkali (%)		
		18 (Sample Ia)	20 (Sample Ib)	24 (Sample Ic)
1	Oxygen delignification 87 psig (O_2_)	✓	✓	-
2	Addition of initial chlorine dioxide (D_0_)	✓	✓	✓
3	Extraction with peroxide (Ep)	✓	✓	✓
4	Addition of second chlorine dioxide (D_1_)	✓	✓	✓
5	Addition of third chlorine dioxide (D_2_)	✓	✓	✓
6	Sulfuric acid administration	-	-	✓

✓: treatment. -: not given treatment.

**Table 4:** Chemical characteristics of whole-empty fruit bunches (WEFB) and stalk-empty fruit bunches (SEFB).

No	Parameter	Analysis Results (%)		Test Method	Literature Study (%)
		WEFB	SEFB		
1	Water	7.66 ± 0.04	8.52 ± 0.11	SNI 087070	<10
2	Ash	3.35 ± 0.01	1.51 ± 0.05	SNI ISO 1762	0.3–1.5
3	Pentosan	27.52 ± 0.11	34.88 ± 0.08	SNI 01-1561	19–25
4	Extractive	7.60 ± 0.17	6.69 ± 0.01	SNI 8401	2–5
5	Lignin	23.17 ± 0.38	20.33 ± 0.08	SNI 8429	20–33
6	Holocellulose	71.59 ± 0.02	80.59 ± 1.59	Wise Method	55.40–76.68
7	Alpha-cellulose	46.62 ± 0.11	48.38 ± 0.68	ASTM D 1103	40–45
8	Solubility in cold water	7.74 ± 0.08	1.07 ± 0.18	SNI 01-1305	1.3–3.5
9	Solubility in hot water	8.19 ± 0.16	2.52 ± 0.21	SNI 01-1305	1.38–7.37
10	1% NaOH solubility	26.91 ± 0.23	27.43 ± 0.12	SNI 14-1838	17.1–23.4

Source: Data of this study.

**Table 5:** Fiber dimensions of whole-empty fruit bunches (WEFB) and stalk-empty fruit bunches (SEFB).

No	SEFB			WEFB		
	Length (mm)	Diameter (mm)	Ratio (L/D) mm	Length (mm)	Diameter (mm)	Ratio (L/D) mm
1	355	0.322	1101.344	159	0.404	393.889
2	345	0.263	1311.787	183	0.314	583.422
3	265	0.252	1050.198	129	0.357	361.682
4	200	0.198	1011.804	147	0.246	597.561
5	232	0.222	1043.478	107	0.272	393.865
6	181	0.216	839.258	134	0.164	815.416
7	255	0.322	792.746	155	0.285	543.224
8	137	0.389	351.884	185	0.241	767.635
9	181	0.346	523.121	125	0.320	390.219
10	272	0.277	980.769	123	0.336	366.435
Average	242.30	0.281	900.639	144.700	0.294	521.335
StDev	70.951	0.062	284.788	25.923	0.068	168.805

Source: Data of this study.

**Table 6:** Dissolving pulp (DP) quality standard based on Indonesian National Standard (SNI) number 938-2017.

No	Parameter	Unit	Requirements
1	Cellulose alpha	%	min. 94
2	S_18_	%	max. 4.9
3	S_10_	%	max. 7.9
4	Extractive content (dichloromethane)	%	max. 0.2
5	Ash content	%	max. 0.15
6	Acid insoluble ash content	ppm	max. 100
7	Calcium	ppm	max. 150
8	Iron content (as Fe)	ppm	max. 10
9	Viscosity (intrinsic)	mL/g	min. 370
10	Viscosity (cupryethylenediamine)	mPa.s or cP	min. 6.2
11	Degrees of brightness	% ISO	min. 88
12	Moisture content	%	max. 12

Source: Data of this study.

**Table 7:** Main analysis results of dissolving pulp (DP) from whole-empty fruit bunches (WEFB).

No	Parameters	Unit	Active Alkali (%)			SNI 938:2017
			18	20	24	
1	Moisture content	%	7.39	7.26	6.51	≤12
2	Alpha cellulose	%	94.17	93.84	94.55	≥94
3	Brightness	% ISO	82.87	81.41	85.43	≥88
4	Kappa number		14.84	14.75	11.24	10–18
5	Cooking yield	%	40.93	37.84	33.37	
6	Bleaching yield	%	91.11	96.43	96.81	
7	Final yield	%	37.29	36.49	32.31	

Source: Data of this study.

**Table 8:** Analysis results of dissolving pulp (DP) on whole-empty fruit bunches (WEFB) fiber with the addition of active alkali 24%.

No	Parameter	Unit	Active Alkali (24%)	SNI 938:2017
1	Moisture content	%	6.51 ± 0.27	≤12
2	Ash content	%	1.80 ± 0.01	≤0.15
3	Acid insoluble silica/ash	mg/kg	11,650 ± 70.71	≤100
4	Alpha cellulose	%	94.55 ± 0.13	≥94
5	S_18_	%	3.07 ±0.11	≤4.9
6	S_10_	%	7.14 ± 0.15	≤7.9
7	Viscosity	cp	7.01 ± 0.01	≥6.2
8	Intrinsic viscosity	mL/g	331.80 ± 0.28	≥370
9	Ca	mg/kg	608 ± 78.09	≤150
10	Fe	mg/kg	354 ± 9.04	≤10
11	Brightness	% ISO	85.43 ± 0.09	≥88
12	Extractive	%	0.82 ± 0.01	≤0.2

Source: Data of this study.

**Table 9:** Analysis results of dissolving pulp (DP) on stalk-empty fruit bunches (SEFB) fibers.

No	Parameter	Unit	Active Alkali (%)		SNI 938:2017
			20	24	
1	Moisture content	%	7.42 ± 0.19	8.99 ± 0.09	≤12
2	Ash content	%	0.41 ± 0.02	0.22 ± 0.01	≤0.15
3	Acid insoluble silica/ash	mg/kg	3100 ± 1.41	1092 ± 2.98	≤100
4	Alpha cellulose	%	89.51 ± 0.06	94.53 ± 0.16	≥94
5	S_18_	%	8.49 ± 0.07	4.56 ± 0.19	≤4.9
6	S_10_	%	18.23 ± 0.01	8.43 ± 0.03	≤7.9
7	Viscosity	cp	15.76 ± 0.04	8.79 ± 0.01	≥6.2
8	Intrinsic viscosity	mL/g	557 ± 0.14	382.1 ± 0.43	≥370
9	Ca	mg/kg	94.50 ± 14.89	82.25 ± 13.63	≤150
10	Fe	mg/kg	24.09 ± 0.66	22.76 ± 2.38	≤10
11	Brightness	% ISO	90.33 ± 0.14	86.36 ± 0.38	≥88
12	Extractive	%	0.46 ± 0.01	0.22 ± 0.01	≤0.2
13	Kappa number		12.01 ± 0.48	5.69 ± 0.21	11–18
14	Brightness	%	90.33	86.36	≥88
15	Cooking yield	%	47.96 ± 0.31	35.71 ± 0.36	
16	Bleaching yield	%	88.35 ± 1.96	94.21 ± 0.16	
17	Final yield	%	42.38 ± 1.22	33.64 ± 0.39	

Source: Data of this study.

## Data Availability

Data available in a publicly accessible repository.
